# α-Synuclein Seeding Activity in Fixed Antemortem Skin Biopsies From Patients With Synucleinopathies

**DOI:** 10.21203/rs.3.rs-10953905/v1

**Published:** 2026-10-11

**Authors:** Zerui Wang, Maria Gerasimenko, Tricia Gilliland, Bailey Bellaire, Todd Levine, Christopher Gibbons

**Affiliations:** The First Affliated Hospital of Guangzhou Medical University; Case Western Reserve University; Case Western Reserve University; CND life science; CND life science; The First Affliated Hospital of Guangzhou Medical University

**Keywords:** α-synuclein, seed amplification assay, RT-QuIC, skin biopsy, Parkinson’s disease, dementia with Lewy bodies, multiple system atrophy

## Abstract

α-Synuclein seed amplification assays can detect seed-competent pathological α-synuclein in fresh and frozen biospecimens, but their performance in fixed antemortem skin biopsies is less well defined. We applied an optimized real-time quaking-induced conversion assay to paraformaldehyde-fixed skin biopsy sections from participants with Parkinson’s disease (PD), dementia with Lewy bodies (DLB), multiple system atrophy (MSA), and controls. Fixed tissue retained measurable α-synuclein seeding activity, although amplification was delayed and the apparent 50% seeding dose was lower than in unfixed or autopsy skin. In this selected proof-of-concept cohort, SAA positivity was observed in 70% of PD, 80% of DLB, 80% of MSA, and 20% of control samples; exploratory ROC analysis yielded an AUC of 0.86. Seeding readouts correlated with Hoehn and Yahr stage but not significantly with MoCA score or pS129 α-synuclein burden. Fixed skin biopsies can therefore provide complementary morphological and functional measures of peripheral α-synuclein pathology.

## Introduction

Synucleinopathies, including Parkinson’s disease (PD), dementia with Lewy bodies (DLB), and multiple system atrophy (MSA), are a group of neurodegenerative disorders characterized by the pathological aggregation of alpha-synuclein (α-syn) protein. These aggregates, forming Lewy bodies and Lewy neurites, are hallmarks of synucleinopathy pathology and play a pivotal role in disease progression [1–4]. Despite advances in understanding these disorders, reliable diagnostic tools for identifying synucleinopathies during life remain elusive, particularly for non-invasive or minimally invasive testing. Diagnosis often relies on clinical evaluation, with definitive confirmation achieved only through post-mortem detection of pathological α-syn aggregates in the brain [5–7].

Ultrasensitive seed amplification assays, such as real-time quaking-induced conversion (RT-QuIC) and related methods, have emerged as powerful tools for detecting the seeding activity of misfolded α-syn aggregates with exceptional sensitivity and specificity. These assays have demonstrated strong diagnostic performance in cerebrospinal fluid (CSF) samples from patients with PD, DLB, and MSA [8–10], and more recently have been extended to peripheral tissues, including skin biopsies [11–14]. The detection of pathologically phosphorylated or misfolded α-syn in the peripheral nervous system—such as within autonomic fibers innervating the colon, salivary glands, and skin—has expanded our understanding of synucleinopathy pathology beyond the central nervous system [15–17].

Evidence suggests that peripheral α-syn pathology may occur early in the disease process and contribute to prodromal manifestations, such as autonomic dysfunction, hyposmia, and REM sleep behavior disorder [18, 19]. In certain conditions, such as pure autonomic failure, peripheral α-syn deposits appear in the absence of overt central nervous system pathology, supporting the concept of a “body-first” subtype within the broader framework of Parkinson’s disease pathogenesis. However, the precise temporal and causal relationship between peripheral and central α-syn aggregation remains an active area of investigation, and interpretations should be made with caution. Nonetheless, these observations collectively highlight the biological plausibility of detecting α-syn pathology in peripheral tissues as a potential biomarker for early or prodromal disease states.

Multiple studies using immunofluorescence and immunohistochemistry have localized α-syn aggregates to autonomic and small unmyelinated nerve fibers in the dermis of patients with PD, DLB, and MSA [17, 20–22]. Such findings have strengthened the rationale for pursuing skin-based biomarkers as a diagnostic tool for synucleinopathies. The accessibility of skin biopsies and the relatively high density of peripheral nerve fibers make the skin an attractive substrate for studying α-syn pathology. Moreover, autonomic and sensory dysfunction linked to cutaneous α-syn accumulation may emerge before the onset of classical motor symptoms, underscoring the diagnostic potential of this approach [21, 23]. Despite these advantages, previous immunostaining-based studies have shown variability in detection rates and moderate sensitivity, which have limited the clinical translation of peripheral biomarkers [3].

A major challenge in peripheral biomarker development lies in the extremely low abundance of pathological α-syn aggregates outside the brain [24–26]. Seed amplification assays, by exponentially amplifying trace amounts of misfolded protein, provide a powerful means of overcoming this limitation. In this study, we optimized the RT-QuIC assay for paraformaldehyde-fixed skin tissues to detect α-syn seeding activity associated with synucleinopathies. Fixed tissues are widely available in clinical and neuropathological repositories and thus provide an invaluable resource for retrospective and comparative studies. By demonstrating the feasibility and sensitivity of α-syn RT-QuIC in fixed skin biopsies from patients with PD, DLB, and MSA, we aimed to establish a robust and quantitative diagnostic framework. Importantly, our findings reveal distinct seeding characteristics across disease types, with particularly strong α-syn seeding activity observed in MSA, suggesting potential utility for disease stratification.

Together, this study provides a proof-of-concept for using RT-QuIC to assess α-syn seeding in fixed skin tissues for broader application in early diagnosis, disease stratification, and monitoring of synucleinopathies.

## Results

### Phosphorylated α-synuclein localizes to cutaneous nerve fibers in fixed skin biopsies

Double-label immunofluorescence demonstrated phosphorylated α-synuclein (p-α-synuclein) deposition within cutaneous nerve fibers. PGP9.5 immunoreactivity delineated nerve fibers as a continuous green fluorescent signal (Fig. 1A), whereas α-synuclein phosphorylated at serine 129 (pS129) showed linear and punctate red immunoreactivity along the same fiber bundles (Fig. 1B). Merged images showed spatial co-localization of pS129 α-synuclein with PGP9.5-positive fibers (Fig. 1C), consistent with localization of pathological α-synuclein within cutaneous nerves.

### α-Synuclein seeding activity is retained in fixed skin biopsy tissue

Using full-length recombinant human wild-type α-synuclein as substrate, α-synuclein seeding activity remained detectable after tissue fixation and processing. Compared with frozen autopsy PD skin, fixed biopsy samples showed delayed amplification and lower maximal Thioflavin T (ThT) fluorescence under the assay conditions used (Fig. 2A).

We next performed endpoint dilution analysis to estimate the 50% seeding dose (SD50). Frozen autopsy PD skin showed a mean LogSD50/mg tissue of approximately 6.3, whereas unfixed biopsied PD skin showed a mean value of approximately 3.3. Seeding activity remained measurable in fixed biopsied PD skin, with a mean LogSD50/mg tissue of approximately 2.4 (Fig. 2B). Recombinant α-synuclein preformed fibrils (5mg/ml) were included as a positive reference and yielded a mean LogSD50/mg of approximately 9.7. These data indicate that a measurable fraction of α-synuclein templating activity persists after fixation. Because the autopsy, unfixed biopsy, and fixed biopsy specimens differed in tissue source and preanalytical handling, however, the quantitative differences among these sample classes should be interpreted descriptively rather than as a direct estimate of the effect of fixation itself.

### Fixed skin biopsies from synucleinopathy cases remain detectable α-synuclein seeding activity

After optimization of the RT-QuIC conditions for fixed biopsy tissue, we analyzed samples from PD (n = 20), DLB (n = 10), MSA (n = 10), and control participants (n = 10). Because the disease samples in this proof-of-concept cohort were selected from biopsies with immunohistochemically confirmed pS129 α-synuclein and sufficient residual tissue, the proportion of SAA-positive samples is reported as a detection rate rather than an unbiased estimate of diagnostic sensitivity. SAA positivity was observed in 14 of 20 PD samples (70%), 8 of 10 DLB samples (80%), and 8 of 10 MSA samples (80%). Two of 10 control samples (20%) were SAA-positive, corresponding to 80% specificity within this selected cohort. The amplification profiles differed across groups: PD samples showed the greatest endpoint ThT fluorescence under the assay conditions used, whereas controls showed the longest lag phase (Fig. 3A–C). Because maximal ThT fluorescence depends on both seed-dependent conversion and the properties of the amplified products, differences in fluorescence amplitude were not interpreted as direct measures of absolute seed abundance. Exploratory receiver operating characteristic analysis based on the SAA fluorescence readout yielded an area under the curve of 0.86 for distinguishing synucleinopathy cases from controls (Fig. 3D). Given the selected nature and modest size of the cohort, this analysis was used to summarize separation between groups rather than to establish clinical diagnostic performance.

### Relationship between fixed-skin seeding readouts, pS129 pathology, and clinical measures

We next explored whether the SAA fluorescence readout was associated with clinical severity or the local burden of phosphorylated α-synuclein. Spearman rank correlation showed a weak inverse association between endpoint ThT fluorescence and Montreal Cognitive Assessment (MoCA) score (ρ = −0.26, p = 0.06), which did not reach statistical significance (Fig. 4A). In contrast, higher ThT fluorescence was associated with more advanced Hoehn and Yahr stage (ρ = 0.58, p < 0.0001; Fig. 4B). A weak positive association was also observed between the pS129 α-synuclein composite score and ThT fluorescence (Spearman ρ = 0.24, p = 0.068; Fig. 4C), although this relationship was not statistically significant. Thus, among the clinical and pathological variables examined, only Hoehn and Yahr stage showed a statistically significant association with the SAA fluorescence readout in the pooled exploratory analysis. The limited correlation between pS129 burden and SAA signal is consistent with the two methods measuring related but non-equivalent properties: immunofluorescence measures the presence and distribution of phosphorylated α-synuclein, whereas SAA measures the ability of seed-competent assemblies to template conversion of recombinant substrate.

Taken together, these findings support the biological relevance of RT-QuIC–detected α-syn seeding activity as a potential correlate of both clinical severity and peripheral α-syn pathology. However, given that this study represents a single timepoint cross-sectional analysis, we cannot infer disease progression from these data. Future longitudinal studies in larger and clinically diverse cohorts will be necessary to determine whether α-syn seeding activity in peripheral tissues may serve as a predictive marker of disease trajectory or cognitive decline.

## Discussion

This study shows that α-synuclein seeding activity can be recovered from paraformaldehyde-fixed antemortem skin biopsies from patients with PD, DLB, and MSA. The main significance of this finding is not simply that pathological α-synuclein can be detected in skin, which has already been demonstrated by both immunostaining and seed amplification assays [10, 12, 13, 27], but that tissue previously fixed for pathological evaluation can still retain measurable α-synuclein templating activity. Previous studies established α-synuclein seeding activity in fresh or frozen skin [10, 12, 13], and seeding activity has also been detected in formaldehyde-fixed α-synuclein-containing tissue [26]. Our findings extend these observations to fixed antemortem biopsy specimens obtained from living participants with several synucleinopathies. This creates the possibility of obtaining both morphological and functional information from the same clinically collected tissue specimen.

The lower apparent SD50 and delayed amplification observed in fixed biopsy samples are biologically plausible consequences of tissue fixation and processing. Aldehyde fixation introduces covalent crosslinks that may restrict conformational flexibility, reduce fibril fragmentation, or limit access of recombinant substrate to seed-competent surfaces. Nevertheless, measurable SD50 values were still detected after fixation, indicating that a proportion of pathological α-synuclein assemblies retains templating competence despite chemical crosslinking. This is consistent with prior work showing that formaldehyde-fixed α-synuclein-containing tissue can remain competent for seed amplification [26].

However, the quantitative differences observed among autopsy skin, unfixed biopsy skin, and fixed biopsy skin should not be attributed solely to fixation. These sample groups differed in tissue source and preanalytical history and were not derived from paired portions of the same specimen. A direct estimate of fixation-induced loss of seeding activity will therefore require paired analysis of adjacent biopsy tissue processed with and without fixation.

An important observation was the limited correlation between the pS129 α-synuclein composite score and the SAA readout. Although both assays interrogate pathological α-synuclein, they measure different properties of the underlying aggregates. Immunofluorescence provides spatial and semiquantitative information about phosphorylated α-synuclein within cutaneous nerve fibers, whereas RT-QuIC measures the ability of seed-competent assemblies to recruit and convert soluble recombinant substrate. Pathological burden and seeding competence therefore need not vary in parallel. This distinction may explain why samples with comparable pS129 deposition can exhibit different amplification profiles and suggests that immunostaining and SAA should be considered complementary rather than interchangeable measurements. In this context, combining anatomical localization with a functional seeding assay may provide a more complete characterization of peripheral α-synuclein pathology than either approach alone.

Differences in amplification kinetics among PD, DLB, and MSA should also be interpreted cautiously. Lag time and maximal ThT fluorescence are influenced not only by seed concentration, but also by aggregate conformation, substrate compatibility, fibril growth properties, and ThT-binding characteristics of the amplified products. Accordingly, higher endpoint fluorescence in one diagnostic group should not be interpreted directly as evidence of a greater tissue burden of pathological α-synuclein. Disease-associated differences in amplification profiles may instead reflect variation in the biochemical or conformational properties of the seed populations present in skin. Endpoint dilution-derived SD50 measurements are better suited to quantitative comparison of seeding dose, and larger disease-specific studies will be needed to determine whether fixed skin retains reproducible seeding signatures that distinguish PD, DLB, and MSA.

The detection of seeding activity in skin also reinforces the view that α-synuclein pathology is not confined to the central nervous system. Phosphorylated and misfolded α-synuclein has been demonstrated in peripheral autonomic structures, including skin, salivary glands, and the gastrointestinal tract [15–17]. These findings have contributed to the hypothesis that, in at least a subset of patients, peripheral α-synuclein pathology may arise early in the disease course and potentially participate in the subsequent spread of pathology toward the CNS. Our data are compatible with the broader concept of peripheral involvement in synucleinopathies, but they do not address the temporal sequence or direction of pathological spread. The present study is therefore more directly informative about the persistence and detectability of peripheral seed-competent α-synuclein than about the origin of disease pathology.

The association between higher SAA fluorescence and more advanced Hoehn and Yahr stage suggests that peripheral α-synuclein seeding characteristics may relate to clinical disease severity. This finding should, however, be regarded as exploratory. The study was cross-sectional, the sample size was limited, and the analysis included disorders with different clinical phenotypes and trajectories. In addition, Hoehn and Yahr stage is primarily a measure of motor impairment and does not represent an equivalent marker of disease severity across PD, DLB, and MSA. The absence of statistically significant associations with MoCA score or the pS129 composite score further argues against interpreting the current SAA readout as a marker of disease progression. Diagnosis-specific analyses and longitudinal sampling will be required to determine whether changes in skin seeding activity track clinical progression or treatment response.

Several limitations should be considered. First, the cohort was relatively small, particularly for DLB, MSA, and healthy controls, resulting in imprecise estimates of detection rate and specificity. Second, disease samples were selected from participants with immunohistochemically confirmed pS129 α-synuclein and available residual tissue. The study cohort was therefore enriched for peripheral α-synuclein pathology and cannot provide an unbiased estimate of diagnostic sensitivity in an unselected clinical population. Third, two control samples were SAA-positive. These results could reflect nonspecific amplification, preanalytical variation, or biologically detectable α-synuclein seeding in individuals without a clinically recognized synucleinopathy; the present study cannot distinguish among these possibilities. Independent repeat testing and longitudinal clinical follow-up would help clarify their significance. Fourth, fixed biopsy, unfixed biopsy, and autopsy samples were not paired, limiting interpretation of the quantitative effect of fixation. Finally, the clinical correlation analyses were exploratory and require confirmation in larger, diagnosis-specific cohorts.

Despite these limitations, the ability to recover α-synuclein seeding activity from fixed antemortem skin biopsy tissue broadens the utility of skin-based α-synuclein biomarkers. Fixed biopsy material can support both anatomical assessment of pS129 α-synuclein and a functional biochemical measurement of seed competence, allowing complementary information to be obtained from the same specimen. This may be particularly useful for retrospective analysis of existing biopsy cohorts and for studies examining the relationship among local pathological burden, seeding behavior, clinical phenotype, and disease-associated α-synuclein conformations. Rather than replacing established pathological or SAA-based biomarkers, fixed-tissue SAA may provide an additional molecular readout that extends the information obtainable from a minimally invasive skin biopsy.

## Conclusion

Our study introduces a novel, sensitive, and minimally invasive method for detecting α-synuclein seeding activity in fixed skin biopsies, paving the way for its application in diagnosing and monitoring synucleinopathies. By widely available tissue samples and amplification techniques, this approach holds promise for advancing personalized medicine in neurodegenerative diseases. Future research should focus on refining the assay, validating it in diverse patient cohorts, and unraveling the pathological significance of peripheral α-syn aggregates, ultimately aiming to improve early detection and intervention strategies for synucleinopathies.

## Materials and Methods

### Participants and skin biopsy specimens

Patient samples and data were obtained from the previously published Synuclein-One trial [27], a prospective, multicenter blinded clinical study of patients with a clinical diagnosis of a synucleinopathy (NCT NCT04700722).[27, 28] Study approval provided by Sterling Institutional Review Board (#8385). Written informed consent was obtained from all study participants. As previously reported, all patients had 3-millimeter punch skin biopsy samples taken from the neck, thigh and distal leg regions. Samples were fixed in 4% paraformaldehyde solution, frozen, and cut into 50-micron thick sections. Tissue was dual immunostained for protein gene product 9.5 (PGP9.5 – a panaxonal marker) and phosphorylated alpha synuclein.[27]^2^ Quantitative measurements of phosphorylated alpha-synuclein were taken from each subject and reported as a single composite score. All subjects underwent standardized neurological examinations including the Montreal Cognitive Assessment (MoCA), the Movement Disorder Society United Parkinson’s Disease Rating Scale (MDS-UPDRS) and Hoehn and Yahr scores[29, 30]. A subset of subjects from the original study with immunohistochemically confirmed phosphorylated alpha-synuclein on skin biopsy also had RT-QuIC completed on the remaining tissue sections (PD (n = 20), DLB (n = 10), MSA (n = 10) and healthy controls (n = 10)).

### Immunofluorescence and Pathological Evaluation

Skin sections were evaluated using dual immunofluorescence for PGP9.5 and pS129 α-synuclein as described in the Synuclein-One protocol [27, 28], PGP9.5-positive nerve fibers were identified in green and pS129 α-synuclein immunoreactivity in red. Co-localization within cutaneous nerve fibers was assessed in merged images. The pS129 α-synuclein composite score used for correlation analyses was obtained from the standardized pathological assessment performed in the parent study.

### Purification of Recombinant α-Synuclein

The purification of recombinant α-synuclein (α-syn) was executed according to previously published methodologies [10, 32]. The BL21 E. coli strain, previously transformed with a plasmid expressing human α-syn, was cultured in Terrific Broth Medium until an optical density of 0.9 was achieved at 600 nm. Cells were then induced using the Overnight Express^™^ Autoinduction System 1 (Sigma-Aldrich) over a period of 24 hours. For purification, the cells were lysed in a high-salt buffer (750 mM NaCl, 10 mM Tris, 1 mM EDTA, and 1 mM phenylmethylsulfonyl fluoride) and then boiled at 100°C for 20 minutes. Following the removal of cell debris, the lysate underwent overnight dialysis in 10 mM Tris, 50 mM NaCl, and 1 mM EDTA before being loaded onto a Superdex 75 column (GE Healthcare Life Sciences). α-Syn-containing fractions were identified via SDS-PAGE and Coomassie blue staining, followed by another overnight dialysis in 10 mM Tris, 25 mM NaCl, and 1 mM EDTA. The protein underwent chromatographic separation using a Hi-Trap Q HP anion-exchange column (GE Healthcare Life Sciences) and eluted using a 0 to 1 M NaCl gradient; α-syn typically elutes around 350 mM NaCl as observed by absorption at 280 nm. SDS-PAGE and Coomassie blue staining were utilized to confirm the purity of α-syn. Finally, purified α-syn was dialyzed into 10 mM Tris and 50 mM NaCl, divided into aliquots, and stored at −80°C until required.

### Preparation of Paraformaldehyde-Fixed Human Skin Homogenate

Human skin tissues were fixed in 4% paraformaldehyde at 4°C. Subsequently, the samples were scraped off into tubes for rehydration, achieved by sequential incubation with decreasing alcohol concentrations (100, 90, 80, and 70% ethanol), and finally 1x PBS, centrifuging at 13000 g for 15 minutes each. Samples were then homogenized with 1x lysis buffer (100 mM NaCl, 10 mM EDTA, 10 mM Tris, 0.5% NaDOC and 0.5% NP-40 with protease inhibitor, pH 7.5) at 5% w/v using a mini beads-beater (Biospec). Homogenates were stored at −80°C until further use. Prior to the RT-QuIC reaction, the homogenates were briefly sonicated for 1 minute in a water bath sonicator (Q700) and then diluted in 0.025% SDS/1X N2 supplement (Gibco) to the desired concentration for RT-QuIC reactions.

### Paraformaldehyde-fixed Real-Time Quaking-Induced Conversion (RT-QuIC) Analysis

The conditions for RT-QuIC reaction were modified from previous versions [33–35]. For α-synuclein, the reaction mix was prepared with 100 mM Phosphate buffer (pH 8.2), 50 mM NaCl, 20 μM ThT, and 6.7 μM protein. In a 96-well plate (Nunc) pre-loaded with six 0.8mm glass beads, 98 μl of reaction mix was added to each well, followed by seeding with 2 μl of skin diluents that had been centrifuged at 5000 g for 5 minutes at 4°C. Plates were sealed with a film (Nalgene Nunc International), and then incubated at 42°C in a BMG FLUOstar Omega plate reader. The incubation process involved 1 minute of double orbital shaking at 400 rpm and 15 minutes of rest for the specified duration. ThT fluorescence measurements (450 ± 10 nm excitation and 480 ± 10 nm emission; bottom read) were recorded every 45 minutes. Four replicate reactions were performed for the same sample dilution, and the average fluorescence values were calculated using data from all replicates. A sample was considered positive if at least half of the replicates exceeded the specified threshold. For quantifying RT-QuIC prion-seeding activity, end-point dilution titrations were used to determine the sample dilution that resulted in positive reactions in half of the replicates (typically 2 out of 4 replicates). This is referred to as the 50% seeding dose, or SD_50_.

### Endpoint dilution and SD50 estimation

Ten-fold serial endpoint dilutions were prepared for each sample, with four replicate reactions analyzed at each dilution. The proportion of positive wells at each dilution was used to calculate the 50% seeding dose (SD_50_) by the Spearman–Kärber method. SD_50_ values for skin samples were normalized to the amount of tissue represented in the seeded homogenate and expressed as LogSD_50_ per mg tissue. No BCA-based protein normalization was applied. Samples without detectable seeding at the highest tested tissue concentration were considered below the assay detection limit for quantitative comparison.

### Statistical analysis

Analyses were performed in GraphPad Prism version 9.5.1. Continuous data are presented as mean ± SD unless otherwise indicated. Student’s t test was used for two-group comparisons. Associations between SAA fluorescence readouts and MoCA score, Hoehn and Yahr stage, and the pS129 α-synuclein composite score were assessed using Spearman rank correlation and are reported as Spearman’s rho (ρ) with two-sided p values. Receiver operating characteristic analysis was used to summarize separation between synucleinopathy cases and controls. Statistical significance was defined as p < 0.05. Because the clinical correlation analyses were exploratory, p values were not interpreted as confirmatory evidence of prognostic association. Statistical significance was assessed using a Student’s t-test with p-value thresholds indicated as follows: * ≤ 0.05, ** ≤ 0.01, *** 0.001, **** 0.0001.

## Figures and Tables

**Figure 1:**
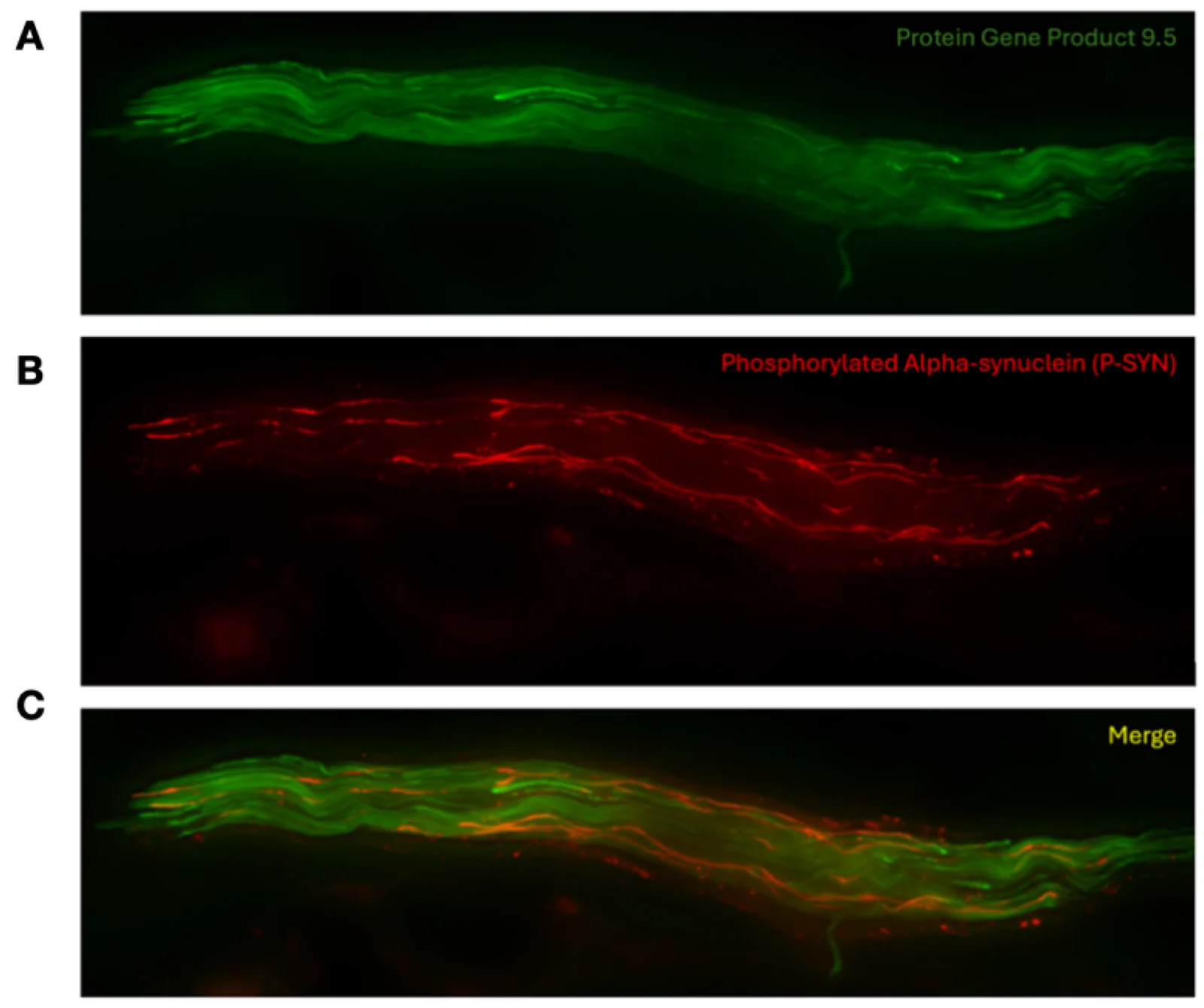
Phosphorylated α-synuclein in cutaneous nerve fibers. Representative double-label immunofluorescence of a fixed skin biopsy section. (A) Cutaneous nerve fibers labeled with the pan-neuronal marker protein gene product 9.5 (PGP9.5; green). (B) Phosphorylated α-synuclein detected with an antibody specific for α-synuclein phosphorylated at serine 129 (pS129; red). (C) Merged image showing spatial co-localization of pS129 α-synuclein with PGP9.5-positive nerve fibers (orange/yellow).

**Figure 2:**
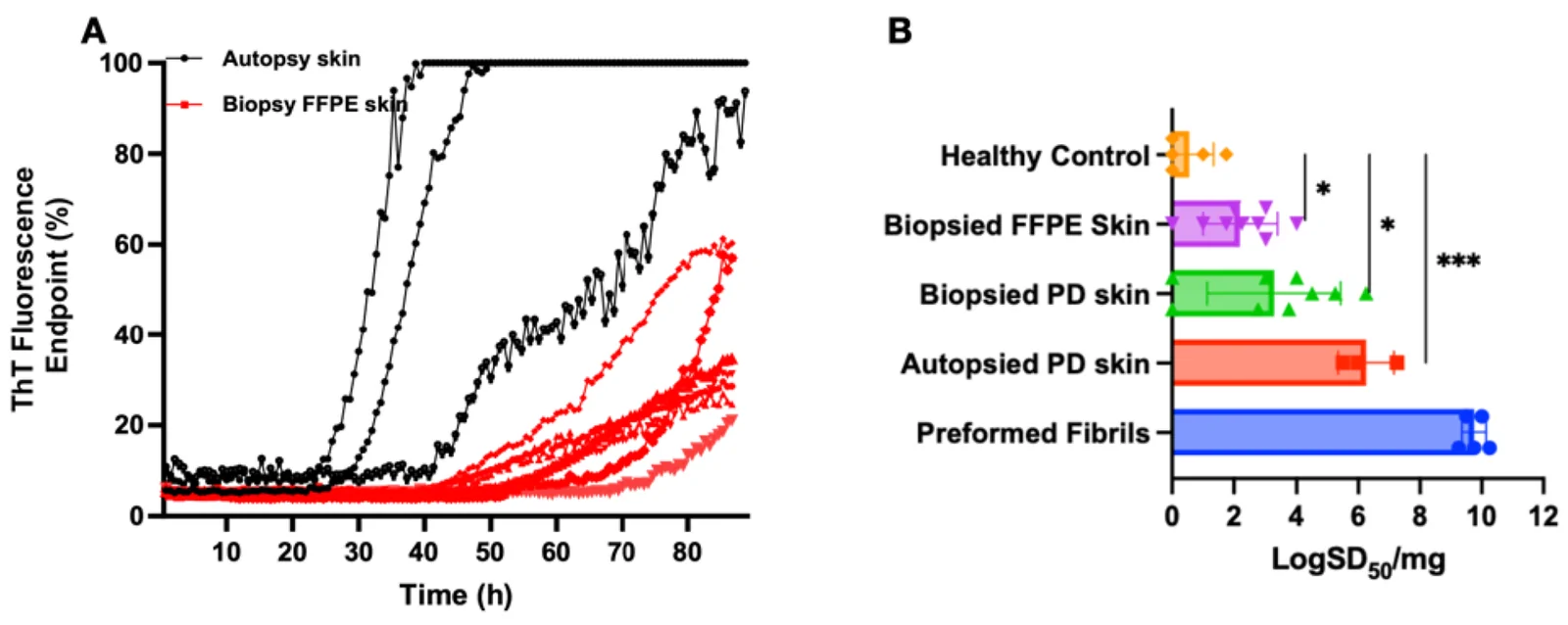
α-Synuclein seeding activity is retained in fixed skin biopsy tissue. (A) Representative RT-QuIC kinetic traces from frozen autopsy PD skin (n = 3) and fixed biopsied PD skin (n = 7). Each trace represents the mean of quadruplicate wells for one subject. (B) Endpoint dilution analysis of α-synuclein seeding activity. Skin seeding activity was calculated using the Spearman–Kärber method and expressed as LogSD50 per mg tissue. Frozen autopsy PD skin showed the highest apparent seeding dose among the tissue groups, followed by unfixed biopsied PD skin and fixed biopsied PD skin. Recombinant α-synuclein preformed fibrils were included as a positive reference.

**Figure 3:**
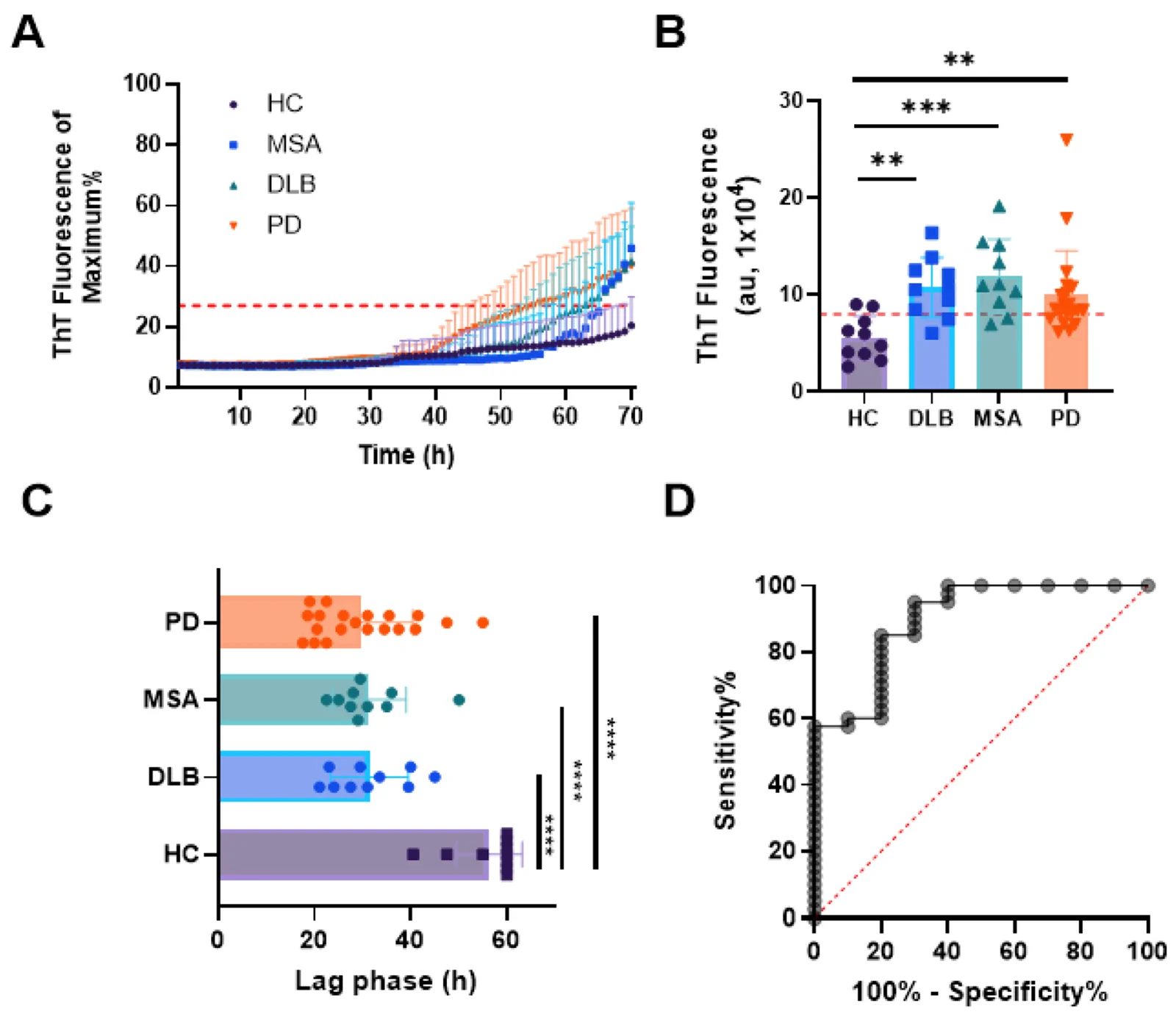
α-Synuclein seeding activity in fixed skin biopsies from synucleinopathy cases and controls. (A) RT-QuIC amplification kinetics for fixed skin samples from PD (n = 20), DLB (n = 10), MSA (n = 10), and controls (n = 10). (B) Distribution of the endpoint SAA fluorescence readout across diagnostic groups. (C) Comparison of lag phase across groups. (D) Exploratory receiver operating characteristic analysis comparing synucleinopathy cases with controls (AUC = 0.86).

**Figure 4:**
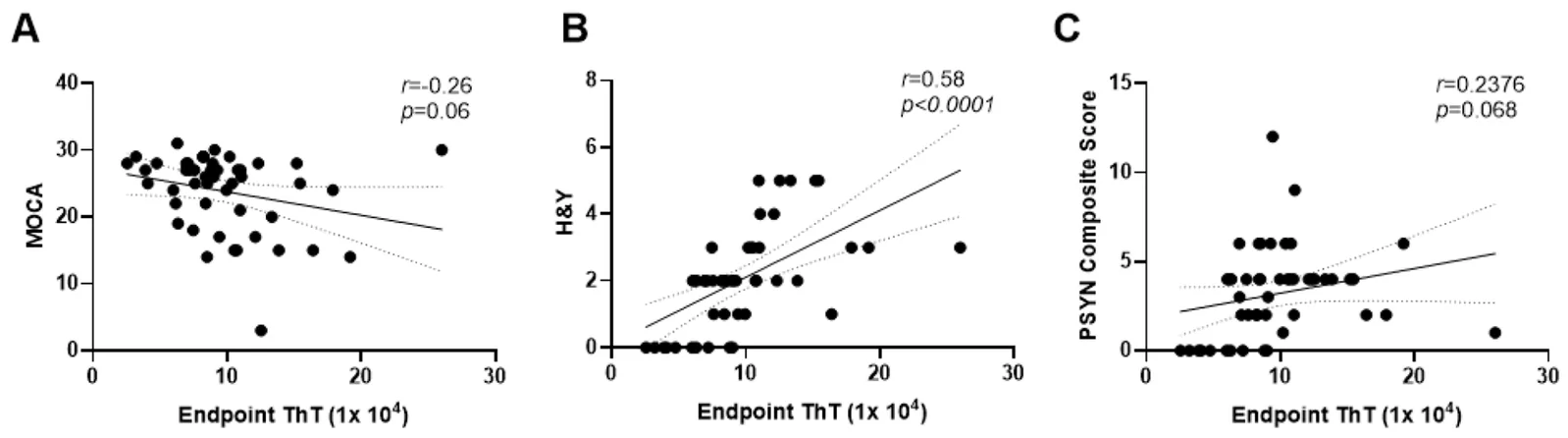
Correlation of seeding activities of fixed skin samples from AD with clinical features. (A) Spearman correlation between endpoint ThT fluorescence and MoCA score (ρ = −0.26, p = 0.06). (B) Spearman correlation between endpoint ThT fluorescence and Hoehn and Yahr stage (ρ = 0.58, p < 0.0001). (C) Spearman correlation between endpoint ThT fluorescence and the pS129 α-synuclein composite score (ρ = 0.24, p = 0.068). Correlation analyses were exploratory and pooled across the analyzed cohort.

**Table 1.** Demographic and clinical features of biopsied cases in different groups

Clinical diagnosis	Number of cases	Sex (M/F)	Age (yrs., mean ± SD)	PSYN Composite Score	SAA Positivity Rate	MOCA (mean ± SD)	Hoehn & Yahr stage (mean ± SD)

Parkinson’s Disease	20	10/10	68.35 ± 9.43	3.25 ± 1.71	70%	26.5 ± 2.98	2.98 ± 2.05

Multiple System Atrophy	10	4/6	66 ± 5.25	4.8 ± 1.99	80%	23.9 ± 4.38	3.4 ± 1.58

Dementia with Lewy bodies	10	9/1	77.1 ± 6.85	4.6 ± 1.67	80%	15.3 ± 5.19	2.6 ± 1.17

Healthy Control	10	4/6	62 ± 11.66	0	20%	27.2±1.99	0

Values are mean ± SD unless otherwise indicated. SAA, seed amplification assay; MoCA, Montreal Cognitive Assessment.

## Data Availability

All data are available in the main text or the supplementary materials.
